# Histone Deacetylase 4 Promotes Osteosarcoma Cell Proliferation and Invasion by Regulating Expression of Proliferating Cell Nuclear Antigen

**DOI:** 10.3389/fonc.2019.00870

**Published:** 2019-09-06

**Authors:** Kun Cao, Hao Wang, Yueyang Fang, Yuan Wang, Lei Wei, Xi Chen, Zheng Jiang, Xiaochun Wei, Yong Hu

**Affiliations:** ^1^Department of Orthopaedics, First Affiliated Hospital of Anhui Medical University, Hefei, China; ^2^Department of Orthopaedics and Department of Surgery, Warren Alpert Medical School of Brown University/Rhode Island Hospital (RIH), Providence, RI, United States; ^3^Shanxi Key Laboratory of Bone and Soft Tissue Injury Repair, Department of Orthopaedics, Second Hospital of Shanxi Medical University, Taiyuan, China

**Keywords:** HDAC4, PCNA, ubiquitination, progression, osteosarcoma

## Abstract

**Background/Aims:** Osteosarcoma (OS) is commonly characterized by lower survival rates and high incidences of local recurrence due to its highly aggressive nature and metastatic tendencies. Studies have shown that histone deacetylase 4 (HDAC4) and proliferating cell nuclear antigen (PCNA) are highly expressed in cancers. Nevertheless, the roles of HDAC4 and PCNA in osteosarcoma (OS) remain unclear. This research aimed to study the expression of HDAC4 and PCNA and their relation to cell proliferation and invasion in human OS.

**Methods:** The levels of HDAC4 and PCNA mRNA and protein were tested in human OS and osteochondroma (OC) tissues. The overexpression and knockdown of HDAC4 in OS cell lines were used to determine the effect of HDAC4 on the expression and degradation of PCNA. The effect of HDAC4 on cell proliferation, invasion and apoptosis was also detected. Additionally, we explored the interaction between HDAC4 and PCNA.

**Results:** The results showed that both HDAC4 and PCNA were increased in human OS tissues. Overexpression of the HDAC4 protein increased the protein level of PCNA, had no effect on the PCNA mRNA level, and decreased the level of ubiquitinated PCNA. We found that overexpression of HDAC4 promoted cell proliferation and invasion and inhibited apoptosis. The opposite effects were observed when HDAC4 was knocked down. The results also showed that HDAC4 could bind to PCNA directly.

**Conclusions:** Our findings suggest that HDAC4 could promote OS cell proliferation and invasion by regulating the expression of PCNA. Thus, our research indicates that HDAC4 may be a potential target for therapy in OS.

## Introduction

Osteosarcoma (OS) is one of the most common primary malignant tumors of bone, and it appears mostly in children and adolescents. The morbidity of OS is moderate. However, its biological characteristics of high aggressiveness and lung metastatic tendencies lead to poor survival, and most patients die of lung metastases (1, 2). At present, the main clinical treatments for patients with OS are still surgery, radiotherapy and chemotherapy. However, traditional treatment is not effective, especially for those who have highly aggressive OS or lung metastases (3, 4). The molecular and functional mechanisms of OS are not clear. Therefore, more research is needed to discover therapeutic targets for a future cure for OS.

Epigenetics is a rapidly emerging field of research in recent years. Epigenetics involves only changes in gene expression and not changes in DNA sequences (5). Increasing evidence has demonstrated that the regulation of epigenetics plays an important role in the proliferation and differentiation of cancer cells. Thus, research on the regulation of epigenetics has attracted increasing attention (6).

In recent years, many studies have revealed that tumourigenesis is closely related to histone acetylation and deacetylation (7). This reversible, dynamic modification is catalyzed by histone acetyltransferases (HATs) and histone deacetylases (HDACs) and is in relative balance under physiological conditions. Once the balance is broken, the activity of HDACs is significantly increased, which could result in an imbalance in the expression of molecules and affect cell proliferation and lead to cell carcinogenesis.

Histone deacetylase 4 (HDAC4) is a member of the classical class IIa histone deacetylase family. Recent studies have shown that HDAC4 has an intimate correlation with tumourigenesis. According to some reports, HDAC4 can regulate the growth and proliferation of cloned cancer cells and is associated with the pathogenesis of hepatocellular carcinoma (8, 9). Thus far, the role of HDAC4 in the pathogenesis of OS is still unclear, and the expression of HDAC4 in OS tissues has not been reported. Hence, understanding the role of HDAC4 in OS cells is of great significance to curing OS in the future.

Proliferating cell nuclear antigen (PCNA) plays an important role in cell proliferation initiation and is a good indicator of cell proliferation (10). Ubiquitination of PCNA may be closely related to the malignant phenotype of certain tumors. Studies have shown that PCNA can be modified by a small ubiquitin-like modifier (SUMO) and then be further modified by ubiquitin (ub), which causes degradation of PCNA (10, 11). In the event of DNA damage, some complexes are recruited to PCNA residues and cause monoubiquitination or polyubiquitination (12). Protein ubiquitination by SUMO is a specific pathway that is subject to strict temporal regulation, and thus, abnormalities in this protein degradation pathway may be associated with malignant tumors (13, 14). Some studies demonstrate that PCNA expression is increased with the malignancy of OS and that decreasing PCNA expression can inhibit the proliferation of OS cells (15, 16). However, the specific mechanisms of PCNA in the development of OS have not been identified, and whether it is related to abnormal degradation through ubiquitination has not been reported.

Until now, the correlation between HDAC4 and PCNA has also not been reported. In previous studies (17), our group found that HDAC4 could increase the expression of PCNA in OS cells. However, the specific mechanism of the relationship is unknown. In this report, we studied whether overexpression of HDAC4 could promote the development of OS by inhibiting the ubiquitination-mediated degradation of PCNA.

## Results

### The Expression of HDAC4 and PCNA in OS and OC Specimens

**Figure 1:**
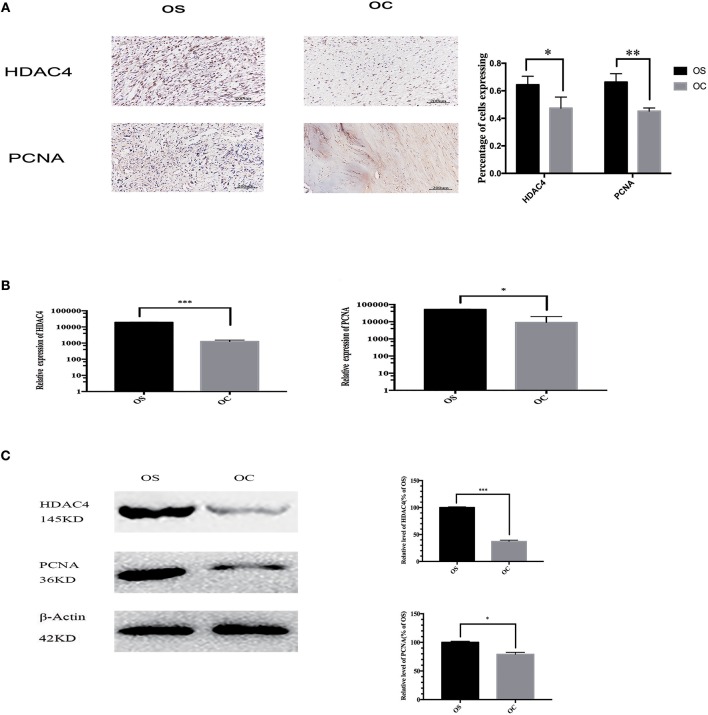
The expression of HDAC4 and PCNA in OS and OC specimens. **(A)** The results of immunohistochemistry showed higher a percentage of cells expressing HDAC4 and PCNA in one OS sample than in one OC sample. **(B)** The mRNA levels of HDAC4 and PCNA in one OS and one OC sample were detected by real-time PCR (^*^P < 0.05, ^**^P < 0.005, and ^***^P < 0.001 by *t*-test). **(C)** Western blotting showed that the expression of both HDAC4 and PCNA was increased in one OS sample compared to that in one OC sample. The internal reference β-actin was used to ensure the same loading quantities of protein samples (^*^P < 0.05, ^**^P < 0.005, and ^***^P < 0.001 by *t*-test). *n* = three independent replicates.

We collected OS and OC tissues from 47 patients and compared the expression of HDAC4 and PCNA in these samples. The results of immunohistochemistry staining demonstrated that the expression of the two proteins was upregulated in OS tissues (Figure 1A). We randomly chose 3 OS and 3 OC tissues to analyse by qRT-PCR and western blotting. We found that the mRNA expression levels of HDAC4 and PCNA detected by qRT-PCR were higher in OS tissues than in OC tissues (*P* < 0.05) (Figure 1B). The HDAC4 and PCNA protein expression levels measured by western blotting were also higher in OS tissues than in OC tissues (*P* < 0.05) (Figure 1C).

### HDAC4 Positively Regulates PCNA in OS Cells

**Figure 2:**
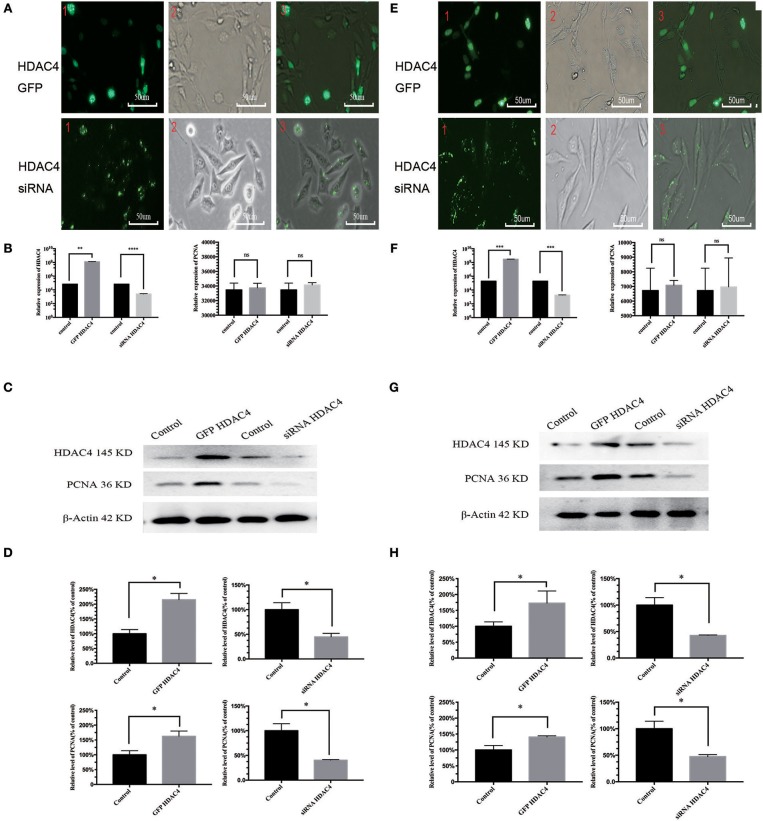
HDAC4 positively regulates PCNA in MG-63 and U2-OS cells. The fluorescence showed the gene transfection efficacy of HDAC4-GFP, HDAC4 siRNA or control in MG-63 **(A)** and U2-OS **(E)** cells. qRT-PCR was used to measure the expression level of HDAC4 and PCNA in MG-63 **(B)** and U2-OS **(F)** cells that had been transfected with HDAC4-GFP, HDAC4 siRNA or control. Western blotting showed the expression levels of HDAC4 and PCNA in MG-63 **(C,D)** and U2-OS **(G,H)** cells transfected with HDAC4-GFP, HDAC4 siRNA or control. ^*^P < 0.05, ^**^P < 0.005, ^***^P < 0.001, and ^****^P < 0.0001 by *t*-test, *n* = three independent replicates. Red 1: fluorescence microscopy image, red 2: microscopy image, red 3: composite image.

We also evaluated the potential relationship between HDAC4 and PCNA. For this purpose, OS MG-63 and U2-OS cells were transfected with HDAC4 siRNA, HDAC4-GFP or control. Transfection efficacy was higher than 90% based on fluorescence microscopy analysis (Figure 2A). The qRT-PCR results showed that HDAC4 siRNA effectively knocked down HDAC4; however, there was no significant difference in PCNA expression (Figure 2B). The qRT-PCR results also showed that cells transfected with HDAC4 over-expression vectors had higher expression levels than the controls, and PCNA expression showed no difference (Figure 2B). The western blotting results indicated that the protein expression levels of HDAC4 and PCNA after transfecting the siRNA were both decreased compared with those of the controls. In addition, the expression level after overexpressing HDAC4 was upregulated for both HDAC4 and PCNA (*P* < 0.005) (Figures 2C,D). The results obtained with the U2-OS cells were the same as those obtained with the MG-63 cells (Figures 2E–H). Our results showed that the regulation of HDAC4 on PCNA protein expression levels was independent of gene transcription levels.

### HDAC4 Expression Level Is Associated With PCNA Ubiquitination, and There Is an Interaction Between HDAC4 and PCNA in OS Cells

**Figure 3:**
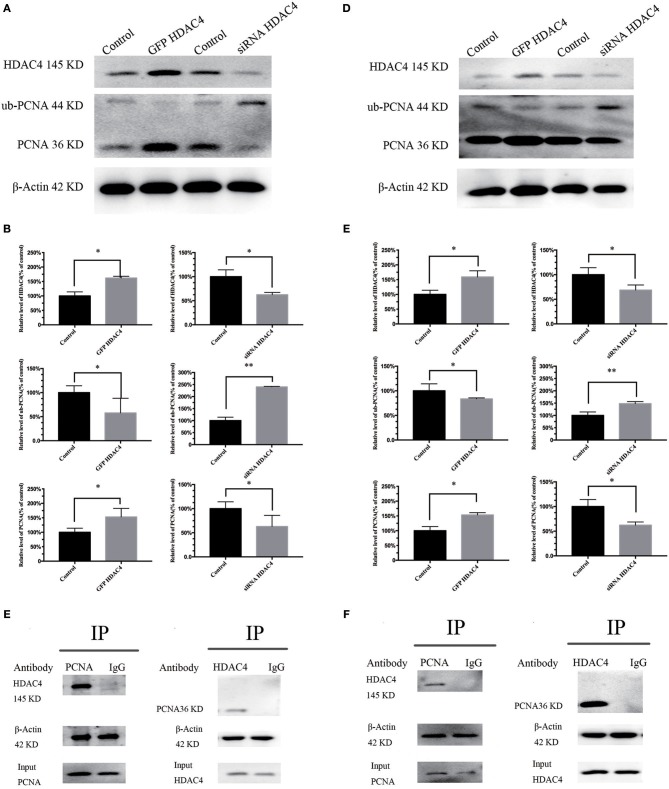
HDAC4 expression is associated with PCNA ubiquitination, and there is an interaction between HDAC4 and PCNA in MG-63 and U2-OS cells. Western blotting showed ubiquitinated PCNA (ub-PCNA) levels in MG-63 **(A)** cells and U2-OS **(D)** cells transfected with HDAC4-GFP or HDAC4 siRNA compared with that in the control. Semi-quantitative densitometry showed the mean concentration of ubiquitinated PCNA after transfection compared with the controls in MG-63 **(B)** cells and U2-OS **(E)** cells (^*^P < 0.05 and ^**^P < 0.005 by *t*-test). The co-immunoprecipitation results showed that HDAC4 was detected in the PCNA immunoprecipitate, demonstrating that HDAC4 was captured by the anti-PCNA antibodies in MG-63 **(C)** cells and U2-OS **(F)** cells. *n* = three independent replicates.

To detect whether HDAC4 is associated with PCNA ubiquitination, we analyzed MG-63 and U2-OS cells transfected with HDAC4 siRNA, HDAC4-GFP or control. The results of western blotting indicated that PCNA ubiquitination was significantly decreased in cells overexpressing HDAC4 compared with that in the controls (P < 0.005). In contrast, silencing HDAC4 increased ubiquitinated PCNA levels compared with those of the control in MG-63 cells (*P* < 0.05) (Figures 3A,B). The results obtained with U2-OS cells were the same (Figures 3D,E).

To investigate the potential possibility of interaction between HDAC4 and PCNA, we prepared OS cells in the period of logarithmic growth without transfection. First, total protein extracts from OS cells were incubated with anti-PCNA antibodies, and then the precipitate was analyzed by western blotting using anti-HDAC4 antibodies. An equal amount of total extract that was not subjected to precipitation was used as a control. Our results showed that HDAC4 was detected in PCNA immunoprecipitates, demonstrating that HDAC4 was captured by the anti-ubiquitinated PCNA antibodies (Figure 3C). Next, the extracts were subjected to a co-IP assay using anti-HDAC4 antibodies, followed by western blot analysis using anti-PCNA antibodies and anti-ubiquitinated PCNA(ub-PCNA) antibodies. The control was prepared as previously described. The results showed that only PCNA was detected in the HDAC4 immunoprecipitate, demonstrating that PCNA was captured by the anti-HDAC4 antibodies (Figure 3C). The results obtained with U2-OS cells were the same (Figure 3F).

### HDAC4 Overexpression or Silencing Is Related to the Proliferation and Apoptosis of OS Cells

**Figure 4:**
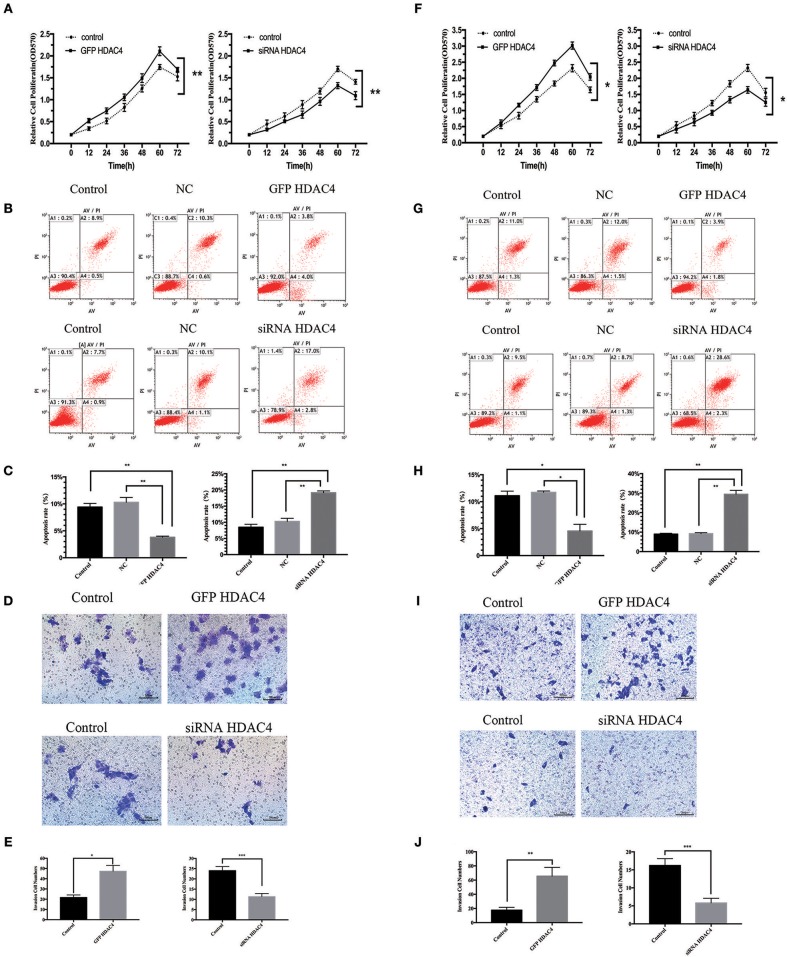
HDAC4 overexpression or silencing is related to proliferation, apoptosis and invasion of MG-63 cells. NC (negative control). An MTT assay was performed to test the proliferation of MG-63 **(A)** cells and U2-OS **(F)** cells transfected with HDAC4-GFP, HDAC4 siRNA or control (^*^P < 0.05, ^**^P < 0.005, and ^***^P < 0.001 by *t*-test). Apoptosis was analyzed by Annexin V-FITC/PI staining in MG-63 cells treated as previously described (^*^P < 0.05, ^**^P < 0.005, and ^***^P < 0.001). HDAC4 overexpression or silencing affects the invasion ability of MG-63 **(B,C)** and U2-OS **(G,H)** cells. The invasion capacities of MG-63 **(D,E)** and U2-OS **(I,J)** cells treated as previously described were assessed using a Transwell assay (^*^P < 0.05, ^**^P < 0.005, and ^***^P < 0.001 by *t*-test). *n* = three independent replicates.

We further studied the influence of HDAC4 on the proliferation and apoptosis of OS cells. MG-63 and U2-OS cells were transfected with HDAC4-GFP, HDAC4 siRNA or control. First, we tested the proliferation of OS cells using an MTT assay. The results demonstrated that the proliferation capacity of OS cells transfected with HDAC4-GFP was significantly increased compared with that of the control group (*P* < 0.005) (Figures 4A,F). Reciprocally, the proliferation of MG-63 cells transfected with HDAC4 siRNA was decreased compared with that of the control (*P* < 0.005) (Figures 4A,F). Second, we assessed apoptosis with Annexin V-FITC/PI staining, which demonstrated that apoptosis was decreased in MG-63 cells transfected with HDAC4-GFP compared with the control cells (*P* < 0.05) (Figures 4B,C), whereas apoptosis was significantly increased in MG-63 cells transfected with HDAC4 siRNA compared with the control cells (*P* < 0.05) (Figures 4B,C). The results obtained with U2-OS cells were the same (Figures 4 G,H).

### HDAC4 Overexpression or Silencing Affects the Invasion Ability of OS Cells

We then studied the effect of HDAC4 on the invasion ability of OS cells. Similarly, MG-63 and U2-OS cells were transfected with HDAC4-GFP, HDAC4 siRNA or control. We analyzed invasion using a Transwell assay. We found that the invasion capacity of cells transfected with HDAC4-GFP was significantly increased compared with that of control cells (*P* < 0.05) (Figures 4D,E), whereas the invasion of cells transfected with HDAC4 siRNA was immensely decreased compared with that of control cells (*P* < 0.001) (Figures 4D,E). The U2-OS cells showed the same result (Figures 4I,J).

## Discussion

HDAC4, which is a member of the class II histone deacetylases, can regulate gene transcription and cell proliferation and plays a crucial biological role in tumourigenesis and metastasis (18, 19). Many studies have indicated that HDAC4 can contribute to the progression of different cancers (20–22). However, HDAC4 has hardly been researched in OS. In our study, we found that HDAC4 expression was higher in OS tissues than in OC tissues (Figure 1). We also demonstrated that overexpressing HDAC4 promoted proliferation and invasion and inhibited apoptosis in OS cells and that silencing HDAC4 had the opposite effects (Figure 4). This observation, for the first time, showed a positive relationship between HDAC4 expression and OS development. Nevertheless, the specific mechanism of positive regulation by HDAC4 in OS cells remains unclear.

PCNA, as the cell proliferation status evaluation index, is uniquely expressed in normal proliferative cells and tumor cells and could facilitate tumourigenesis and proliferation of a variety of tumors, including OS (23–26). Consistent with HDAC4, we also found that PCNA expression was upregulated in OS tissues compared with OC tissues (Figure 1). Our results further showed that the protein expression level of PCNA was positively correlated with that of HDAC4; however, the mRNA expression level of PCNA was not correlated with that of HDAC4 (Figure 2). Therefore, these observations implied that PCNA might be a downstream target of HDAC4, and we speculated that there might be a positive correlation between HDAC4 and PCNA in OS. Taken together, these results show that HDAC4 overexpression could promote OS cell proliferation and invasion and inhibit apoptosis by upregulating PCNA expression.

Above all, the importance of HDAC4 in the development and progression of OS may be realized through upregulating the expression level of PCNA. From the previously described results, we can demonstrate that regulation may occur at the posttranslational stage but not at the transcription stage. Analysis of the levels of PCNA ubiquitination, which is one of the posttranslational modifications (PTM) (27), by co-IP assay demonstrated that HDAC4 could directly interact with PCNA in OS cells (Figure 3). Our results initially suggest that the two proteins have a direct interaction with each other.

Ubiquitination is ubiquitous in eukaryotic cells. Ub is a small molecule protein, and the sequence is highly conserved. Ub can be covalently connected to target substrates at their lysine residues (28–30). Ubiquitination is sequentially catalyzed by the following series of enzymes: ub-activating enzyme (E1), ub-conjugating enzyme (E2) and ub-ligase enzyme (E3) (20). Similar to other proteins, PCNA can be modified by ub molecules at one Lys residue (monoubiquitination) or multiple Lys residues (polyubiquitination) (31–33). Some studies have shown that the ub-proteasome system (UPS) mediates most of the eukaryotic intracellular protein degradation (5, 34, 35). The 26S proteasome, one of the components of the UPS, is a proteolytic complex and has the ability to recognize ubiquitinated protein substrates, resulting in protein degradation (35, 36). Thus, once PCNA is ubiquitinated, it can be identified by the 26S proteasome for degradation, and PCNA expression is subsequently decreased. We assume that binding of HDAC4 to PCNA conceals the ubiquitin-binding domains (UBDs) of PCNA and then reduces the formation of ub-PCNA and reduces PCNA degradation. To further demonstrate our hypothesis, we have begun research, and the next step is to demonstrate that HDAC4 regulates PCNA ubiquitination by acting on the Lys164 site through techniques such as site-directed mutagenesis.

In conclusion, our results indicated that HDAC4 and PCNA were upregulated in OS. HDAC4 overexpression by HDAC4-GFP promoted proliferation and invasion and inhibited apoptosis in OS cells through resistance to PCNA ubiquitination. Nevertheless, the specific mechanism of the combination of HDAC4 and PCNA is unclear. Further studies are needed to clarify the mechanism. Our studies demonstrated that HDAC4 may be an effective target for OS therapy.

## Materials and Methods

### OS and OC Samples

OS tissues and OC tissues were obtained from the patients who underwent surgical resection in our department (for OS samples, *N* = 24, 14 males, 10 females, average age 15.38 ± 4.98 years [mean ± standard deviation (SD)], range 9–26 years; for OC samples, *N* = 23, 15 males, 8 females, average age 18.10 ± 4.98 years, range 12–30 years). OS and OC tissues were all diagnosed as having pathological evidence. The locations of all OS tissues were 10 cases in the distal femur, 11 cases in the proximal tibia and 3 cases in the proximal humerus. The research was authorized by the Institutional Review Board at the First Affiliated Hospital of Anhui Medical University (approval ID: 20170017) and had received informed consent from all the patients.

### Cell Culture

The MG-63 and U2-OS cell lines were obtained from Shanghai Institutes for Biological Sciences, Chinese Academy of Sciences. The OS cells were cultured in Dulbecco's modified Eagle medium (DMEM) containing 10% fetal bovine serum (FBS, Invitrogen), 2 mM L-glutamine, 100 IU/ml penicillin and 100 μg/ml streptomycin and maintained at 37°C in humidified air with 5% CO_2_. We replaced the medium every 2–3 days and subcultured the cells until the cell confluence reached 90%. The cells were kept in a logarithmic growth state.

### DNA Constructs and Small Interfering RNAs (siRNAs)

HDAC4-GFP and control vector (pEGFP) were obtained from Dr. A. R. Means (37). The cells were digested and then placed in 6-well plates so that the cell density at the time of transfection could reach 30 to 50% confluence. After 1 day, cultures were transfected with HDAC4-GFP and control vector (50 nM) for overexpressing HDAC4. To knock down HDAC4, and siRNA that was designed and compounded by GenePharma (Company, Shanghai, China) was used (5′-AAUGCAGUGGUUCAGAUUCTT-3′). The positive control was GAPDH (5′-UGACCUCAACUACAUGGUUTT-3′). The cells were transfected with HDAC4 siRNA, positive control siRNA (5 μg) and negative control siRNA (5 μg). The transfection was carried out by using Lipofectamine 2000 (Invitrogen) according to the manufacturer's protocol, and cells were further cultured for 24 or 48 h for qRT-PCR or western blot analysis, respectively, before harvesting. Fluorescence microscopy was used to count live GFP-positive cells after transfection for 24 h to detect the transfection efficiency. Similarly, 6 h after transfection, carboxyfluorescein (FAM) was detected by fluorescence microscopy.

### Western Blotting and *in vitro* Ubiquitination Assays

Total protein samples were extracted from OS and OC tissues and from OS cells that were transfected with HDAC4-GFP or HDAC4 siRNA after transfection for 48 h by using cell lysis buffer (Thermo Scientific Pierce IP Lysis Buffer). The protein concentration was measured with a BCA Protein Assay Kit (Pierce). Fifty micrograms of protein per sample was used in sodium dodecyl sulfate–polyacrylamide gel electrophoresis (SDS-PAGE) and electroblotted onto polyvinylidene fluoride (PVDF) membranes. The membranes were blocked with 5% skim milk in Tris buffered saline with Tween 20 (TBST) at 37°C. After 1 h, the membranes were incubated with primary antibodies against HDAC4 (1:1,000, Abcam ab12172) and PCNA (1:500, Santa Cruz Biotechnology sc-25280) at 4°C overnight on a shaker. The next day, secondary antibodies were used to stain the membranes at 37°C for 1 h. The secondary antibodies were horseradish peroxidase (HRP)-conjugated goat anti-rabbit IgG (1:10,000, Santa Cruz sc-2004) and anti-mouse IgG (1:10,000, Santa Cruz sc-2005). The detection was performed by using an enhanced chemiluminescence (ECL) kit.

The *in vitro* ubiquitination assays were performed as described above. When total protein samples were extracted from OS cells after transfection for 48 h, ubiquitinated PCNA was detected by SDS-PAGE and probed with primary antibodies (1:1,000, Cell Signaling Technology D5C7P).

### Quantitative Real-Time Reverse Transcription PCR (qRT-PCR)

Total RNA was extracted from samples and cells transfected with HDAC4-GFP or HDAC4 siRNA after transfection for 48 h by using Trizol reagent (Invitrogen, USA). The RNA was reverse transcribed into cDNA using the PrimeScript™ RT reagent Kit (Takara, Dalian, China). qRT-PCR was performed using SYBR Premix Ex Taq™ II (Takara, Dalian, China) with a LightCycler480 System (Roche Diagnostics) according to the manufacturer's protocols. The sequences of primers for qRT-PCR were: HDAC4, forward 5′-GCCTGGGAGCACTGCCCCTCCACGCACA-3′ and reverse, 5′-CTTGTTCATCTGCAGTTGCTGCTGC-3′; PCNA, forward 5′-ATGTTCGAGGCGCGCCTGGTCCA-3′ and reverse, 5′-CTGGTGAGGTTCACGCCCATG-3′; and GAPDH, forward, 5′-AGAAGGCTGGGGCTCATTTG-3′ and reverse, 5′-AGGGGCCATCCACAGTCTTC-3′. The qPCR procedures were as follows: 30 s at 95°C for initial denaturation, 40 cycles of denaturation at 95°C for 5 s, annealing for 30 s at 64°C (HDAC4) or 67°C (PCNA), and a melting curve profile at 60°C for 30 s. All reactions were run in triplicate.

### Immunohistochemistry

Immunochemical staining was carried out by using the Biotin-Streptavidin Horseradish Peroxidase (HRP) Detection Systems (ZSGB-BIO, SP-9000). Immunohistochemistry (IHC) was applied to test the expression of HDAC4 and PCNA in OS and OC, respectively. The tumor samples were obtained from surgeries and underwent diagnostic biopsies in the pathology department. These tissues were formalin-fixed, embedded in paraffin, sectioned at 5 μm widths and mounted on glass slides. Paraffin sections were deparaffinized conventionally and rehydrated with an ethanol series. Citrate was applied to retrieve antigens, and tissue sections were processed by 3% hydrogen peroxide to eliminate the influence of endogenous peroxidase. The tissue sections were blocked in normal sheep serum and incubated with anti-HDAC4 (1:200, Abcam ab12172) and anti-PCNA (1:500, Santa Cruz Biotechnology sc-25280) primary antibodies at 4°C overnight. The following day, sections were treated with biotinylated secondary antibodies and streptavidin labeled with HRP. The slices were subjected to coloration in 3,3′ diaminobenzidine (DAB) and sequentially counterstained with haematoxylin. The staining results were determined by microscopy. The positive expression of HDAC4 and PCNA was defined as brown-yellow granular deposits around the nucleus, while that of not-expressed was showed as negative. Moreover, the quantification was automatically performed by using analysis software (Image-pro plus, version 6.0). The statistics of each tissues were performed in five randomly selected fields and the error bar was determined based on several data in each group. The results were showed and compared based on the percentage of positive cell number in each group.

### Cell Proliferation Assay

The proliferation assay was performed by using 3-(4,5-dimethylthiazolyl-2)-2,5-diphenyltetrazoliumbromide (MTT; Sigma M2128). The OS cells that had been transfected for 24 h were seeded onto 96-well culture plates in triplicate at a density of 5,000 cells/well and cultured for 0, 12, 24, 36, 48, 60, and 72 h. During the 3-day culture period, 10 μl MTT (5 mg/ml) solution was added to each well at intervals of 12 h, and the plates were cultured for another 4 h. Next, 100 μl of DMSO was added to each well and mixed adequately on the shaker to solubilize the dark blue crystals. The intensity of the cells was measured colorimetrically at a wavelength of 570 nm on a microplate reader.

### Cell Invasion Assay

Cell invasion was assessed by the 24-well Transwell assay (Corning 3422) kit according to the manufacturer's protocols. The Transwell inserts (8 mm pore size) were covered with 50 μl of chilled, diluted BD Matrigel basement membrane matrix (Corning 356234) and incubated at 37°C for 1 h until gelling occurred. After 24 h of transfection, OS cells were digested with trypsin/EDTA (0.25%) and resuspended in 200 μl of serum-free DMEM. Then, 5,000 cells were added to the upper chamber. Five hundred microlitres of DMEM containing 20% FBS were placed in the lower chamber. The plate was incubated at 37°C in a humidified atmosphere with 5% CO_2_ for another 24 h. After that, the penetrating cells on the underside of the membrane were stained in 0.5% crystal violet for 15 min. The stained cells were counted and photographed under a microscope. Each experiment was performed in triplicate.

### Cell Apoptosis Assay

Cell apoptosis was detected by using an Annexin V-FITC/PI apoptosis detection kit (BB-4101-50T) according to the manufacturer's instructions. The cells (10^6^/ml) that had been transfected were collected from 6-well plates by trypsinization without EDTA and washed in cold PBS twice. A total of 400 μl of Annexin V binding buffer was used to resuspend the cells. Five microlitres of Annexin V-FITC was added to the suspension liquid, which was then incubated for 15 min at 4°C away from light. Then, 10 μl of propidium iodide (PI) was added to the cells, and they were incubated for 5 min at 4°C in the dark. Cells were measured by flow cytometry, and the apoptosis results were analyzed by FlowJo7.6.5. Each experiment was carried out in triplicate.

### Co-immunoprecipitation

Co-immunoprecipitation (co-IP) was performed to examine the interaction of HDAC4 and PCNA by using a Pierce™ Co-IP Kit (Pierce 26149). The procedures were performed following the manufacturer's recommendations. Briefly, HDAC4 and PCNA antibodies were added to the AminoLink Plus Coupling Resin for immobilization. The resin was washed in coupling buffer and wash solution prior to co-IP. The cultured cells were lysed in lysis buffer on ice. Cell lysates were centrifuged (12,000 g, 5 min, 4°C), and the supernatant was harvested for co-IP. Then, the supernatant was pre-cleared with control agarose resin, and the flow-through was saved and added to the immobilized antibodies for the co-IP. Next, prey protein mixture was added to the resin and incubated with gentle mixing at 4°C overnight. The second day, samples were washed in lysis/wash buffer and centrifuged. Then, elution buffer was added to the resin, and the flow-through was collected. Sample buffer was added to the sample, which was heated at 100°C for 5 min and then immunoblotted.

## Data Availability

All data sets generated for this study are included in the manuscript.

## Ethics Statement

The research was authorized by the Institutional Review Board at the First Affiliated Hospital of Anhui Medical University (approved ID:20170017) and had received informed consent from all the patients.

## Author Contributions

KC participated in the design of study and revised the manuscript. HW drafted of the manuscript and performed the statistical analysis. YF and YW prepared the OS and OC specimens, carried out the histological experiment, and help to perform the statistical analysis. LW conceived of the study and revised the manuscript. XC carried out the cell culture, molecular biology studies, and co-immunoprecipitation. ZJ prepared the DNA constructs, carried out the transfections, and ubiquitination experiment. XW helped to design the study and revised the manuscript. YH conceived of the study, participated in its design and coordination, and helped to draft the manuscript. All authors read and approved the final manuscript.

### Conflict of Interest Statement

The authors declare that the research was conducted in the absence of any commercial or financial relationships that could be construed as a potential conflict of interest.
